# Pilot and Feasibility Test of a Mobile Health-Supported Behavioral Counseling Intervention for Weight Management Among Breast Cancer Survivors

**DOI:** 10.2196/cancer.5305

**Published:** 2016-05-09

**Authors:** Lisa M Quintiliani, Devin M Mann, Marissa Puputti, Emily Quinn, Deborah J Bowen

**Affiliations:** ^1^ Boston University Department of Medicine Section of General Internal Medicine Boston, MA United States; ^2^ Boston University Department of Medicine Section of Preventive Medicine and Epidemiology Boston, MA United States; ^3^ Boston University School of Public Health Data Coordinating Center Boston, MA United States; ^4^ University of Washington School of Medicine Department of Bioethics & Humanities Seattle, WA United States

**Keywords:** telemedicine, survivors, breast neoplasms, health behavior, body weight

## Abstract

**Background:**

Health behavior and weight management interventions for cancer survivors have the potential to prevent future cancer recurrence and improve long-term health; however, their translation can be limited if the intervention is complex and involves high participant burden. Mobile health (mHealth) offers a delivery modality to integrate interventions into daily life routines.

**Objective:**

The objective of this study was to evaluate the effects of a one-group trial with a pre-post evaluation design on engagement (use and acceptability), physiological (weight), behavioral (diet and physical activity), and other secondary outcomes.

**Methods:**

The 10-week intervention consisted of mHealth components (self-monitoring of selected diet behaviors via daily text messages, wireless devices to automatically track weight and steps) and 4 motivational interviewing–based technology-assisted phone sessions with a nonprofessionally trained counselor. Participants were overweight breast cancer survivors who had completed treatment and owned a smartphone. Weight was measured objectively; diet and physical activity were measured with brief self-reported questionnaires.

**Results:**

Ten women participated; they had a mean age of 59 years (SD 6), 50% belonged to a racial or ethnic minority group, 50% had some college or less, and 40% reported using Medicaid health insurance. Engagement was high: out of 70 days in total, the mean number of days recording steps via the wristband pedometer was 64 (SD 7), recording a weight via the scale was 45 (SD 24), and responding to text messages was 60 (SD 13); 100% of participants completed all 4 calls with the counselor. Most (90%) were very likely to participate again and recommend the program to others. Mean weight in pounds decreased (182.5 to 179.1, mean change −3.38 [SD 7.67]), fruit and vegetable daily servings increased (2.89 to 4.42, mean change 1.53 [SD 2.82]), and self-reported moderate physical activity increased in metabolic equivalent of task (MET) minutes per week (2791 to 3336, mean change 545 [SD 1694]).

**Conclusions:**

Findings support the conduct of a fully powered trial to evaluate the efficacy of mHealth as a feasible intervention modality for breast cancer survivors. Future research should employ accelerometer-based physical activity assessment and consider development of an all-in-one app to integrate devices, messaging, and educational content and other mHealth approaches to support behavioral counselors conducting weight management interventions.

**Trial Registration:**

ClinicalTrials.gov NCT02387671; https://clinicaltrials.gov/ct2/show/NCT02387671 (Archived by WebCite at http://www.webcitation.org/6hGEuttbZ).

## Introduction

Owing to multiple factors including improved treatment, the 5-year survival rate in the United States for women with breast cancer for 2003-2009 is 90%, up from 75% in 1975-1977 [1]. There are >3.1 million female breast cancer survivors in the United States [1]. Increasing attention is now being focused on how to increase quality of life, prevent future cancer recurrence, and reduce risk of chronic diseases, such as cardiovascular disease and diabetes [2].

For people who have already completed treatment and are either disease free or whose cancer is stable, the 2012 Nutrition and Physical Activity Guidelines for Cancer Survivors from the American Cancer Society recommend the following: (1) achieving a healthy weight; (2) moderate-vigorous physical activity of 150 minutes per week and strength training 2 times per week; and (3) eating a diet that is high in vegetables, fruits, and whole grains [2]. However, translation of these findings into population-wide, inexpensive, sustainable programs has to date proven largely unrealized. Key questions remain in terms of how interventions should be optimally designed for maximum effectiveness and reach to the entire cancer survivor population. This is particularly true for populations that face health disparities, those who belong to low-income or racial/ethnic minority groups, as these populations often experience less favorable cancer outcomes and higher rates of obesity compared with other populations [3,4].

Mobile health (mHealth) involves the use of any mobile technology, such as mobile phones and wireless sensors, to deliver and share personalized health information [5]. Mobile health holds immense promise to deliver behavioral interventions that are embedded into individuals’ daily routines, that are highly personalized to individuals’ behaviors, health conditions, and daily routines, and have the potential to reach diverse populations. Smartphone ownership is now higher among black, non-Hispanic (70%) and Hispanic (71%), populations compared with white (61%) populations [6]. In concordance with the promise of mHealth intervention modalities, an increasingly large body of literature now exists that has been examined in multiple systematic reviews covering particular mHealth strategies [7,8] (eg, apps, text messaging) and health topics [9,10] (eg, weight, physical activity, nutrition). A systematic scoping review focused on weight management published in late 2015 revealed that half of the 457 articles reviewed were published within the past 2 years [10]. Bennett and colleagues [11] also reviewed the use of electronic health (eHealth) interventions for weight management among racial/ethnic minority populations and found that interventions featuring more advanced features of eHealth technology and the use of mHealth technologies are needed. Although more research is needed to summarize and recommend best practices, intervention designers can use information from these reviews to help inform the design of future interventions, including which mHealth strategies to select, how to implement them, and how to combine human behavioral counselors with mHealth components.

Although there is much that can be learned from this body of literature that can be applied to the optimal design and development of interventions, there are very few published studies using technology (mobile or Web-based) to deliver interventions to cancer survivors. This is important because cancer treatment can result in a range of circumstances that affect diet and physical activity behaviors of cancer survivors, including changes in taste and smell, physical discomfort such as lymphedema, and changes in social support structures [2]. A systematic review conducted by Goode et al [12] analyzed print, telephone, and Web-based interventions for diet, physical activity, or weight management among cancer survivors. Of 27 studies, 3 were delivered using the Web (1 of which was via the social media platform Facebook) and none were delivered via text message. In addition, none of the studies specifically targeted minority race or ethnic groups. The review concluded with support for broad-reach methods, specifically telephone based, and the need to integrate newer technologies, such as texting and mobile technologies, to deliver interventions with potential for broad reach to diverse populations of cancer survivors. Although some studies have since been published [12] or are in development [13] that have some mHealth components such as texting or use commercial weight loss applications [14], published studies evaluating interventions with multiple mHealth components for weight management and related behaviors among cancer survivors are limited.

Given the body of evidence for the importance of lifestyle behaviors for cancer survivors and the increasingly large literature on mHealth interventions, the time is ripe to deliver mHealth interventions to adult cancer survivors. Our approach is to use multiple mHealth strategies to augment a human counselor-delivered behavioral intervention to address weight management-related behaviors. The purpose of this study was to evaluate a weight management mHealth intervention for breast cancer survivors on the following feasibility and preliminary efficacy outcomes: engagement (use and acceptability); physiological (weight); behavioral (diet and physical activity); and psychological and other outcomes (self-efficacy, perceived stress, social support, sleep, loss of control over eating, fatigue). The selection of these outcomes served to provide a detailed picture of the effects of the intervention directly both on weight and weight-related behaviors and factors that influence these behaviors. The feasibility data served to help the research team decide if this intervention approach is suitable for further testing and refinement in future studies [15].

## Methods

### Study Design

We conducted a one-group pilot study with a pre-post evaluation design to evaluate the feasibility and preliminary efficacy of a human counselor–delivered behavioral intervention incorporating multiple mHealth strategies targeting weight management behaviors among breast cancer survivors (ClinicalTrials.gov NCT02387671). The protocol was approved by the Boston University Medical Center Institutional Review Board.

### Formative Feedback

Before the start of the pilot study, we conducted individual interviews with 4 women from our target population to obtain feedback on mHealth intervention design features. Informed consent was obtained from participants at the start of the interview. Interview participants had a mean age of 62.3 years (SD 7.5) and body mass index (BMI) of 33.5 kg/m^2^(SD 5.0); 1 (25%) reported being Hispanic, 2 (50%) black or African American, and 1 (25%) white. Suggestions for features of an mHealth intervention included providing educational/culturally relevant resources, offering information on opportunities for social support (ie, recommending local support groups), and encouraging the use of mobile phone apps and programs to search the Internet for more information. Concerns included participants believing recalls of food intake could be inaccurate and maintaining an appropriate number of interactions between the counselor and participants. Additionally, the need for a thorough orientation to any mHealth strategies (devices, apps, the agenda for counseling sessions) was noted. We refined our intervention approach in response to the findings.

### Recruitment and Participants

Recruitment methods included contacting individuals on a university-maintained registry of people interested in research, postings on a university website for those looking for research studies, postings on university-wide emails, recontacting individuals from the interviews described above, announcements at the hospital-based breast cancer survivor support group, posting paper flyers, and posting notices on Craigslist and in a local newspaper. Through all methods, individuals were asked to call or email the study team to request further information. When the individual contacted the study team, she was screened for eligibility. Eligibility criteria were 18 years of age or older, able to speak and read English, female, 2 years or more since breast cancer diagnosis (self-reported) and 6 months or more since end of cancer treatment (surgery, radiation, or chemotherapy), self-reported overweight or obese (BMI greater than 25 kg/m^2^, as assessed by self-reported height and weight), be an owner of an Android or iOS-platform smartphone, and have WiFi at home. Exclusion criteria included contraindications for physical activity, pregnancy, presence of a pacemaker or other internal medical device, and medical conditions (dementia, active cancer, anorexia) or any other condition in the opinion of the study team deemed to make the participant unsuitable for inclusion in the study. For eligible individuals, a staff member then scheduled an in-person baseline study visit at our research office space at an academic medical center. At this visit, a staff member measured their height and weight, asked whether they would be willing to share Fitbit account information (log-in/password) with the study team, and observed their ability to navigate programs on a smartphone to verify eligibility. Individuals who had a BMI of 25.0 kg/m^2^or greater according to measured height and weight, were willing to share Fitbit account information, and appeared able to navigate programs on their smartphone (from the research staff members' perspective) were eligible to participate. We chose these eligibility criteria in order to compose a sample that would be similar to a sample targeted in a future, larger-scale, randomized controlled efficacy trial, yet also be feasible to obtain within the parameters of a small-scale pilot study (for example, it was determined that it would not be feasible to provide smartphones to individuals who did not already own one). This sample also served to target breast cancer in early survivorship phases or later such that cancer treatment-related physical effects have decreased for most women [16].

Eligible participants were asked to provide their informed consent to participate and were enrolled into the intervention and asked to complete a paper-and-pencil baseline questionnaire. Participants were recontacted 10 weeks after baseline to complete a follow-up paper-and-pencil questionnaire. Participants received US $20 for completing each questionnaire and received the wristband pedometer and scale devices to keep.

### Intervention

After enrollment, participants engaged in a counselor-delivered intervention with multiple mHealth components [17] over 10 weeks (Textbox 1). Participants engaged in self-monitoring nutrition behaviors; automatic (ie, passive) weight and behavioral monitoring via a scale and wristband pedometer; and received technology-assisted phone counseling from a behavioral health counselor.

**Description of intervention components.**

Intervention component

1. Self-monitoring

Daily text messages sent to ascertain participants’ dietary intake immediately after enrollment and for the next 10 weeks. Each message was the same and contained 5 questions that prompted a yes or no response or a numerical value. Responses from the messages were recorded and conveyed to the health counselor to assist in the counseling sessions. Messages corresponded to content in the telephone counseling sessions.

Questions:

- Did you eat more than one high-calorie snack?
- Did you eat food from a restaurant or fast-food place?
- Did you drink more than one sugary drink?
- Did you eat until you were uncomfortably full?
- How many servings of fruits & vegetables did you eat?

2. Automatic weight and behavioral monitoring

*Weight scale*

Participants were asked to record their body weight daily using Fitbit Aria [18,19].

*Wristband pedometer*

Participants were asked to wear the wristband daily to track their steps and hours of sleep by wearing the Fitbit Flex wristband. Weight, steps, and sleep recordings were synchronized through WiFi (weight) or via cellular Bluetooth (steps & sleep) connections to the participants' Fitbit app installed on their mobile phone.

3. Technology-assisted counseling

*Counselor training*

A behavioral health counselor conducted telephone sessions with participants. The counselor underwent training in study-specific protocols (eg, recording phone calls) and motivational interviewing techniques, such as viewing and discussing a series of 4 training videos, practicing and receiving feedback on counseling with a structured call guide. Before interacting with participants, the study director rated a recorded telephone session between the counselor and a volunteer for attaining a sufficient level of motivational interviewing spirit and empathy (eg, asking permission, supporting the participant and not confronting or giving advice). The coach had a bachelor’s degree in nutrition.

*Counseling session topics and ordering*

Participants engaged in 4 phone calls with the study counselor, one every other week. The first and second calls focused on physical activity, sleep, and fatigue. The third and fourth calls focused on 2 out of 4 possible nutrition topics chosen by the participant. The 4 nutrition topics were sugary beverages, fruits and vegetables, snacking, and cooking/preparing meals/eating out. This approach was intended to bring about small decreases in daily energy intake by making small daily behavioral changes, such as substituting no-calorie beverages for sugary beverages. This “small changes” approach has demonstrated efficacy in multiple populations, including overweight or obese adults [20] and multicultural socioeconomically disadvantaged adults [21,22].

*Counseling session content*

The counselor used a structured, yet flexible, guide to conduct the sessions that followed principles of motivational interviewing [23], the Social Contextual Model [24], and was adapted from a guide used in a previous study [25]. The guide was built in Excel and used embedded logic to flow from section to section. The calls included 6 sections: introduction (introduce the topic of the call, review privacy information); provide feedback on current behavior compared with recommendations, obtain information on participant’s behavior (eg, what type of physical activity she enjoys); assess importance & confidence in changing the behavior; assess influences on their behavior (eg, finances, stress, family/friends/neighborhood); assess motivation to change behavior; if motivated, conduct collaborative goal setting, and if not, prompt discussion of what it would be like to change). In each subsequent call, the counselor checked in about what was discussed in the previous call. Participants’ data collected during self-monitoring were used to guide the counseling sessions. All calls were recorded.

*Resources*

The counselor had a list of weblinks for resources around each topic area (such as sleep, fatigue, fruits and vegetables, and so on) The resources were compiled from sources that were both reputable and user-friendly. Examples included choosemyplate.gov, mayoclinic.org, and cdc.gov. Resources were sent to the participants if the participant requested them.

*Asynchronous messages*

During interim weeks between calls, the counselor maintained contact with the participants through 3 asynchronous text messages per week to monitor their progress in the study. The messages served several purposes:

- The counselor would monitor whether participants were tracking behaviors (self-monitoring and automatic weight and behavioral monitoring). If participants were not tracking all behaviors at least 5 days per week, the counselor would try to resolve any issues with tracking (eg, device difficulties, confusion on how or what to track). If they were meeting the 5-day-per-week target, the counselor encouraged them to keep up the good work.
- The counselor would check in on any goals the participant had set during the counseling calls (eg, “Hi, it looks like you have not yet reached your goal of walking 10,000 steps 7 days per week. Do you have any questions about this goal? Have you been experiencing any difficulties?” or “Just checking in on the goal you set to plan out your meals every Sunday evening. How has that been going? Have you experienced any successes? Any difficulties?”).
- The counselor would send a message to the participant 2 days before each counseling call as a reminder of the upcoming call.
- The participants and counselor also used asynchronous messages as needed to ask/answer questions, comments, or requests for information.

### Measures

Our measures comprised both feasibility (engagement and acceptability) and outcomes (physiological, behavioral, and secondary variables), because it is important to both demonstrate the feasibility of the intervention approach and emulate the evaluation approach of a future larger-scale efficacy trial.

#### Engagement and Acceptability

We collected data on number of calls completed, duration of calls, number of responses received to text messages, and valid days of wearing the wristband pedometer and recording a weight on the scale. Daily step counts of <100 and >50,000 were considered invalid. We also asked open- and closed-ended questions on the appeal of the intervention, perception of the number of calls received, perceptions of setting and meeting health goals, how likely they would be to participate again, and suggestions for improving the intervention.

#### Evaluation Outcomes

##### Physiological

Weight and height were measured using a protocol in which participants removed shoes or footwear, outer garments, and so on and stood with their back against a wall [26]. The same procedure was used at 10 weeks. Height measurements were recorded to the nearest ¼ inch, rounding down [27]. Height was measured at baseline only.

##### Behavioral

Diet was measured by an 18-item food frequency questionnaire, the PrimeScreen, which has been compared for reliability and validity against a full-length food frequency questionnaire and biomarkers [28]. Correlation coefficients for comparability between dietary components of the PrimeScreen and a full-length food frequency questionnaire range from .36 for other vegetables to .82 for eggs and for nutrient estimates range from .48 for folate, .58 for fiber, to .59 for saturated fat. Correlation coefficients for comparability between PrimeScreen and biomarkers were .33 for vitamin E and .43 for both beta-carotene and lutein/zeaxanthin. Participants indicate the frequency with which they eat each food, with 5 response category options, ranging from less than once a week to twice or more per day. Foods were then grouped into categories: fruits and vegetables, 6 items; whole grains, 1 item; red and processed meats, 2 items; whole fat dairy foods, 1 item; and high calorie, 3 items. A composite diet score was calculated, with a score from 0 (worst) to 100 (best) assigned for intake from each of the 5 food categories and then averaged [29].

Sugary beverage intake was evaluated via the 15-item Beverage Questionnaire (BEVQ-15) [30], which assesses frequency of past-month consumption of common sugary drinks including sweetened juice drinks, soda, and energy drinks. Of note, 100% fruit juice is not included as a sugary drink. The BEVQ-15 has shown adequate reliability and validity with 4-day food intake records (Spearman r value = .673 for grams of total sugar-sweetened beverages).

Fast-food intake was assessed via a 1-item question: “In the past 7 days, how many times did you eat fast food? Include meals eaten at work, at home, or at fast food restaurants, carryout or drive-through, such as food you get from Dunkin Donuts, McDonald’s, Panda Express, or Taco Bell,” which was based on a question derived from a large population-based survey [31]. Response options were as follows: less than once per week, once per week, 2-4 times per week, nearly daily, and twice or more per day.

Physical activity was measured using the International Physical Activity Questionnaire (IPAQ) [32,33]. This tool provides an internationally relevant measure of physical activity, which has undergone extensive validity and reliability testing. The IPAQ covers all areas of moderate and vigorous physical activity in everyday life, with questions in regard to job-related physical activity; transportation physical activity; housework and family care physical activity; recreation, sport, and leisure time physical activity; and time spent sitting.

##### Psychological and Other Secondary Variables

Self-efficacy was assessed separately for fruit and vegetable intake and physical activity, asking the participants to rate their confidence that they can perform these behaviors under a variety of circumstances [34]. We used the 4-item Perceived Stress Scale [35] (sample question: “In the last month, how often have you felt that you were unable to control the important things in your life?”), with response options ranging from 0 (never) to 4 (very often). Social support was assessed using the question “How much can you rely on family or friends for support and encouragement?” with answer options a lot, somewhat, and not at all [36]. Sleep was evaluated using the question “How often during the past 4 weeks did you get enough sleep to feel rested upon waking up?” with response options never, rarely, sometimes, often, and very often [37]. Perceived loss of control over eating was evaluated using the validated 7-item Loss of Control over Eating Scale-Brief, with 5 response options ranging from 1 (never) to 5 (very often) [38]. Fatigue was evaluated using a scale of 0-10 with 0 being “no fatigue” ranging up to 10 as fatigue “as bad as you can imagine” [39,40].

### Statistical Analysis

All surveys and measurements for outcomes were conducted in person and collected on paper. Surveys were then entered in duplicate into REDCap [41] by two individuals and compared for accuracy. Data were examined descriptively using frequencies, means, and medians. Analysis was conducted using SAS version 9.3 (Cary, NC).

## Results

### Participant Flow Through the Study

In response to our recruiting efforts, 27 individuals were screened for eligibility. Fourteen were excluded for not meeting the eligibility criteria; the most frequent reasons for being ineligible were not having home WiFi and/or a smartphone (n=9) and not being overweight/obese (n=3). Three individuals were eligible but declined to participate, because of perceived need for more assistance with using the devices, perceptions that the intervention would offer a prescribed diet and exercise program, and not being able to enroll until a later date. Therefore, 10 participants were enrolled. Recruitment methods for enrolled participants were hospital-based breast cancer support groups (n=4), the patient registry (n=3, of whom 2 gave formative feedback), hospital-wide email (n=2), and the newspaper advertisement (n=1). All participants completed both the baseline and follow-up surveys.

### Participant Characteristics

Characteristics of participants are listed in Table 1. Approximately half reported belonging to a minority race/ethnic group and 40% reported financial limitations as reflected by being covered by Medicaid insurance or receiving food assistance benefits within the past 2 years. The majority accessed the Internet on their mobile phone but less frequently used their mobile phone for health-related purposes. Self-reported breast cancer stage was early or 0-I (60%) and stage II or above (40%). Mean self-reported years since diagnosis was 7.1 (SD 4.0).

**Table 1:** Participant characteristics.

Characteristic		N=10
Age in years, mean (SD)		58.6 (6.1)
**Ethnicity/race, n (%)**		
	Hispanic white	1 (10)
	Non-Hispanic black	3 (30)
	Non-Hispanic white	5 (50)
	Other	1 (10)
**Highest level of education, n (%)**		
	High school graduate/GED^a^or lower	2 (20)
	Some college/university	3 (30)
	College/university graduate or higher	5 (50)
**Work for pay, n (%)**		
	Yes	6 (66.7)
	No	3 (33.3)
**Type of insurance, n (%)**		
	Medicaid (ie, public insurance) only or in combination	4 (40)
	Private insurance	5 (50)
	Medicare (ie, public insurance for older adults) & private	1 (10)
**Delayed taking medication due to cost, n (%)**		
	Yes	2 (20)
**Household receives food stamps, n (%)**		
	Yes	4 (40)
**Always had enough money to buy food, n (%)**		
	Yes	9 (90)
**Use the Internet at least occasionally, n (%)**		
	Yes	10 (100)
**Send or receive email at least occasionally, n (%)**		
	Yes	10 (100)
**Access the Internet on a mobile handheld device, n (%)**		
	Yes	9 (90)
**Use mobile phone to download apps, n (%)**		
	Yes	9 (90)
**Have apps to track health, n (%)**		
	Yes	4 (40)
**Receive text updates or alerts about health or medical issues, n (%)**		
	Yes	0 (0)
**Social support**		
	A lot	5 (50)
	Somewhat	5 (50)
	Not at all	0 (0)

^a^GED=General Education Diploma

### Engagement and Acceptability

Out of 70 opportunities (7 days a week × 10 weeks) to record self-monitoring and automatic behavioral monitoring data, mean number of responses was 60 (SD 13), median 64 (range 24-68) for responding to text messages; 64 (SD 7) for recording a step measurement, median 52 (range 3-67); 45 (SD 24) for recording a weight measurement, median 67 (range 52-70); and 43 (SD 19) for recording a sleep measurement, median 47 (range 9-63).

**Figure 1:**
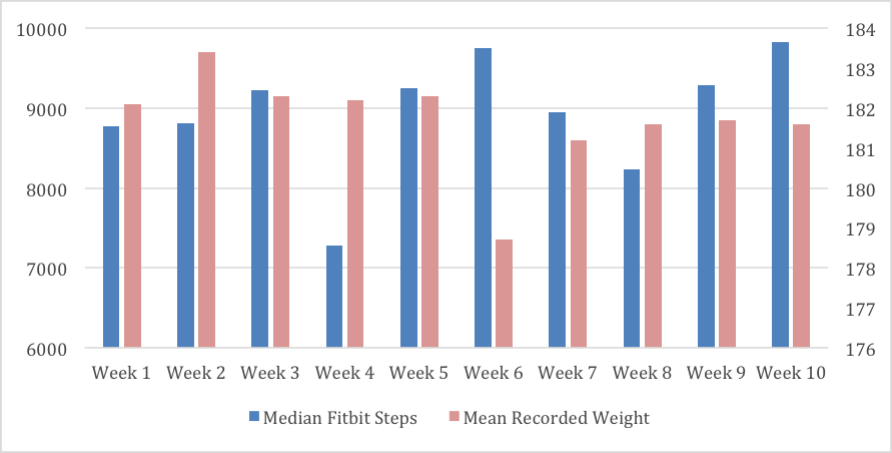
Weekly tracked data for steps and weight recorded via the wristband pedometer and scale tracking devices.

All participants completed all 4 counseling calls. Mean duration of calls 1 to 4 was 29 (SD 9), 22 (SD 11), 28 (SD 14), and 24 (SD 13) minutes, respectively. Of 20 nutrition-related calls completed (2 per participant), there were 8 calls about fruits and vegetables, 6 about cooking, 5 about snacking, and 1 call about sugary drinks. To illustrate the data collected during the intervention, median number of recorded steps and mean weight are presented in Figure 1.

For acceptability, 9 participants reported setting health goals during the last 3 months and all participants reported meeting some (n=8) or all (n=2) of their personal goals. All participants rated the calls as very helpful in setting personal goals to change their health habits and felt the number of calls was “just right.” Whereas 2 participants responded that the number of text messages/emails from their counselor was “too many,” the other 8 felt the number was “just right.” Nine of 10 participants responded that it is “very likely” that they would participate again or recommend the program to others. However, 7 of 10 participants responded that it is “somewhat unlikely” or “not at all likely” that they would participate again if they had to pay for the program.

Written feedback included participants’ difficulty with using the devices (including seemingly erratic weight and sleep readings) and desire for self-monitoring diet behaviors in a more streamlined fashion. Participants noted the calls and the wristband pedometer and scale devices were helpful in setting and achieving goals.

### Evaluation Outcomes

As listed in Table 2 , there were beneficial changes in physiological and behavioral outcomes, including weight, nutrition behaviors (daily servings of fruits and vegetables and the composite diet score), and physical activity. Those reporting “often” or “very often” to feeling rested upon waking in the past 4 weeks increased from 5 at baseline to 7 at follow-up. Other variables showed minimal changes (such as self-efficacy) or nonbeneficial changes (such as intake of sugar-sweetened beverages). For fast-food consumption, 6 stayed at the same response, 2 increased from < 1 time per week to 1 time per week, 1 decreased from once per week to < 1 time per week, and 1 skipped this question at baseline. Beneficial changes were also reported for perceived fatigue, loss of control eating, and perceived stress.

**Table 2:** Change in behavioral, psychological, and other outcomes from baseline to 10-week follow-up.

Outcome		Baseline mean (SD) median (range)	Follow-up mean (SD) median (range)	Change mean (SD) median (range)
Weight, pounds		182.5 (24.9)	179.1 (23.4)	−3.38 (7.67)
**Diet behaviors, servings/day**				
	Fruits & vegetables	2.89 (1.79)	4.42 (1.91)	1.53 (2.82)
	Whole grains	0.30 (0.31)	0.32 (0.30)	0.03 (0.33)
	Whole fat dairy	0.34 (0)	0.31 (0)	−0.03 (0.28)
	Red meat	0.51 (0.42)	0.62 (0.76)	0.11 (0.92)
	High calorie	0.36 (0.31)	0.51 (1.07)	0.15 (0.86)
Diet composite score		60.16 (9.19)	66.91 (8.80)	6.76 (13.31)
Sugar-sweetened beverages, fluid ounces		8 (7)	13 (15)	5 (13)
Low physical activity, MET-minutes/week^a^		1967 (3189) 830 (0-10,584)	3076 (2685) 2473 (0-8262)	1108 (3636) 1029 (−6552 to 8064)
Moderate physical activity, MET-minutes/week^a^		2792 (4475) 660 (0-14,175)	3336 (4422) 1755 (0-14,160)	545 (1694) 345 (−3090 to 3360)
Vigorous physical activity, MET-minutes/week^a^		1776 (4103) 0 (0-13,200)	2568 (3751) 1080 (0-11,040)	792 (5565) 0 (−10,320 to 10,560)
**Self-efficacy**				
	Fruits & vegetables	3.1 (0.6)	2.9 (0.6)	−0.3 (0.6)
	Physical activity	3.0 (1.1)	2.9 (1.0)	−0.1 (0.8)
Fatigue		4.4 (2.1)	2.6 (1.6)	−1.8 (0.8)
Loss of control eating		1.9 (0.8)	1.4 (0.5)	−0.5 (0.7)
Perceived stress		5.1 (3.3)	4.7 (3.2)	−0.4 (3.3)

^a^MET=Metabolic Equivalent of Task

## Discussion

This mHealth-supported behavioral counseling intervention for weight management was feasible to implement, as demonstrated by high levels of engagement with the intervention components (self-monitoring, automatic behavioral monitoring, and counseling phone sessions) and high levels of acceptability with intervention components. In our study, out of 70 opportunities to answer self-monitoring text messages, the mean number of responses was 60, or 86%. Our findings compare favorably with other reported engagement outcomes in the literature. For example, in a study among overweight or obese women from racial/ethnic minority groups, one intervention component included daily text messages prompting self-monitoring with an accompanying feedback message. Among 26 intervention group participants, the adherence rate for responding to the message was 49% (SD 28) [18]. In another intervention using a wearable pedometer (the clip-on Fitbit) among women, mean number of days of wear-time was 106 out of 112 days (94%) [42], which is comparable with our data in which out of 70 opportunities to log a step count via Fitbit wristband pedometer, the mean number of times that participants recorded their steps was 64, or 91%. Taken together, our study has comparable outcomes with other research using mHealth strategies for weight or weight-related behaviors, such as physical activity. It is possible that high levels of engagement with intervention components are the result of the integration of mHealth into participants’ daily lives, allowing for simpler and more frequent self-monitoring.

In addition, there were several beneficial physiological, behavioral, and other variables. Notably, weight change decreased by a mean of 3.38 pounds, which reflects a 2% loss of baseline weight. Although this is lower than the generally accepted clinically meaningful weight loss level of 5%, our intervention was of a shorter duration (10 weeks) and of a moderate intensity level that may be able to be sustained over the long term. Combined with changes in diet and physical activity as well as changes in other variables such as fatigue and sleep, participation in the intervention led to multiple beneficial changes that could be further examined in a larger trial.

About half of our participants were reflective of a population facing health disparities (ie, low income or belonging to a racial/ethnic minority group). Others have examined the use of mHealth strategies among health disparity populations. For example, Smith and colleagues [43] examined the preferences of African American breast cancer survivors for lifestyle modification and found that peer-led sessions and incorporation of support groups would be important components of intervention strategies. The integration of human-based intervention components such as counselor-delivered phone calls with technology-related approaches was also supported by the weight management study conducted among breast cancer survivors by Spark and colleagues [44]. We ultimately decided to involve human coaching because it is not yet established that fully automated eHealth/mHealth interventions have comparable efficacy with interventions that utilize human counselors [11]. Thus, future directions for mHealth interventions for breast cancer survivors from health disparity-facing groups could investigate optimal ways of integrating human-based components, such as behavioral counseling, into mHealth-based interventions. The scalability of this approach can be broadened as community health workers/patient navigators are increasingly integrated into health care systems [45] and can support telephone-based delivery of behavioral interventions. This approach can become even more widely implemented as devices become more available across population groups (eg, among low-, medium-, and high-resourced groups) and data from these devices become better integrated with electronic medical records.

Limitations to our pilot study design include a lack of a control group and a small sample size. Although it is appropriate for sample sizes for pilot studies to be based on practical considerations based on recruitment and budgetary limitations [46], a larger sample size may have expanded our capacity to generate feasibility data. In addition, our eligibility criteria requiring ownership of a smartphone and home WiFi is also a limitation in that it may have led to bias in our sampling design [47]; our criteria may have excluded members of our target population (for example, women from low-income groups without access to home WiFi). These design decisions were made to enable the functionality of the weight scale (which relied on a WiFi connection) and because providing smartphones was beyond the financial resources of the study. However, in future work, we will select newer devices that do not rely on WiFi connectivity, and examine the option of providing lower-cost smartphones to those who do not own one. Another limitation is the lack of objective assessment of physical activity at baseline and follow-up time points via accelerometry. Our self-reported data on physical activity likely reflect an overestimation of physical activity, yet may still be useful in exploring the direction of change from baseline to follow-up time points. Similarly, the data on steps/day as measured by the wristband pedometer during the intervention period also reflected moderately high levels of physical activity. Moreover, other research has shown that the Fitbit Flex wristband pedometer can underestimate step count in treadmill walking and running [48]. Taken together, future studies may consider eligibility criteria in which participants have lower levels of physical activity upon entry to the study. Future research will also systematically capture cancer stage and treatment details from the medical record.

In conclusion, our findings demonstrate that a pilot test of an mHealth-supported behavioral counseling intervention conducted among breast cancer survivors was feasible and demonstrated some positive physiological and behavioral changes. Future work could examine this intervention approach in a larger study, powered to detect significant changes in weight, and further investigate optimal ways to integrate behavioral counseling with mHealth strategies.

## Abbreviations

BEVQ: Beverage Questionnaire
BMI: body mass index
IPAQ: International Physical Activity Questionnaire
mHealth: mobile health
